# Outbreak of Candidozyma auris: a challenge for the infection control team

**DOI:** 10.3205/dgkh000663

**Published:** 2026-07-09

**Authors:** Heera Hassan, Anil Sathyadas, Aravind Reghukumar, Maramparambil Chami Sathyabhama, Jyothi Rajahamsan, Manjusree Shanmugham, Sindhu Moni Sunanda, Priya Krishnan Kutty Nair Chandrika, Jayalekshmi Jayageetha

**Affiliations:** 1 Government Medical College, Thiruvananthapuram, Kerala, India

## Abstract

Between March 15 and April 5, 2025, an outbreak of *Candidozyma auris*, a multidrug-resistant fungal pathogen, was detected in the intensive care unit of a tertiary care center in Thiruvananthapuram, India. Five patients were affected—three with bloodstream infections and two with asymptomatic colonization. Interventions included patient screening, cohorting, enhanced hand hygiene, environmental cleaning, and disinfection of shared equipment. *C. auris* was identified using MALDI-TOF, but antifungal susceptibility testing could not be performed. The probable index case was a colonized patient with prolonged fluconazole exposure. Lapses in hand hygiene were the likely mode of transmission; environmental cultures were negative. No new cases occurred two months after implementation of infection control measures.

The outbreak highlights the need for early species-level identification of non-albicans Candida, improved laboratory capacity, regular staff training, and strict infection control practices. It underscores the importance of preparedness and rapid response to fungal threats in critical care settings.

## Abstract (de)

Zwischen dem 15. März und dem 5. April 2025 wurde in der Intensivstation eines tertiären Versorgungszentrums in Thiruvananthapuram ein Ausbruch von *Candidozyma (C.) auris* festgestellt. Fünf Patienten waren betroffen, drei mit Blutstrominfektionen und zwei mit asymptomatischer Besiedlung. Zu den Maßnahmen gehörten Patienten-Screening, Kohortierung, verstärkte Händehygiene und desinfizierende Flächenreinigung einschließlich benutzter Medizinprodukte.* C. auris* wurde mittels MALDI-TOF identifiziert; eine Antimykotika-Empfindlichkeitsprüfung konnte aufgrund fehlender standardisierter Protokolle nicht durchgeführt werden. Der wahrscheinliche Indexfall war ein kolonizierter Patient mit längerer Fluconazol-Exposition. Die Übertragung erfolgte wahrscheinlich durch mangelnde Händehygiene, weil Umgebungsuntersuchungen negativ waren. Zwei Monate nach Einführung der Infektionskontrollmaßnahmen traten keine neuen Fälle auf.

Der Ausbruch unterstreicht die Notwendigkeit einer frühzeitigen Identifizierung von Nicht-Albicans-Candida auf Artenebene, verbesserter Laborkapazitäten, regelmäßiger Mitarbeiterschulungen und strenger Infektionskontrollmaßnahmen.

## Introduction

Between March 15-April 5 of 2025, an outbreak of *Candidozyma (C.) auris* was detected in an intensive care unit (ICU). Five patients were affected, with 3 infections and 2 colonizations. 

The ICU is a 10-bed unit catering to critically ill patients from medical, surgical, and trauma specialties. Standard IPC protocols in the ICU included hand hygiene adherence monitoring, contact precautions for MDR organisms, and daily audits of environmental cleaning and disinfection. 

## Methods

### Detection of the outbreak

On 15/03/2025, *C. auris* was isolated from a bloodstream culture of a ventilated patient with acute necrotising pancreatitis and septic shock. Over the following 21 days, 4 additional patients in the critical care unit tested positive via blood and/or urine cultures. An alert was generated by the microbiology lab and reported to the IPC team. *C. auris* was identified using MALDI-TOF. Antifungal susceptibility testing could not be performed due to lack of standardized protocols. 

### Case definition

A confirmed case was any patient admitted to ICU from March 3,2025 with laboratory-confirmed *C. auris* from any sterile or non-sterile site. A probable case was any patient admitted to ICU from March 3, 2025 with laboratory-confirmed non-albicans Candida from any sterile or non-sterile site. A colonized case was any patient in the same unit with positive surveillance swab or non-sterile culture (e.g., axilla/groin). 

## Results

### Epidemiological summary

**Figure 1:**
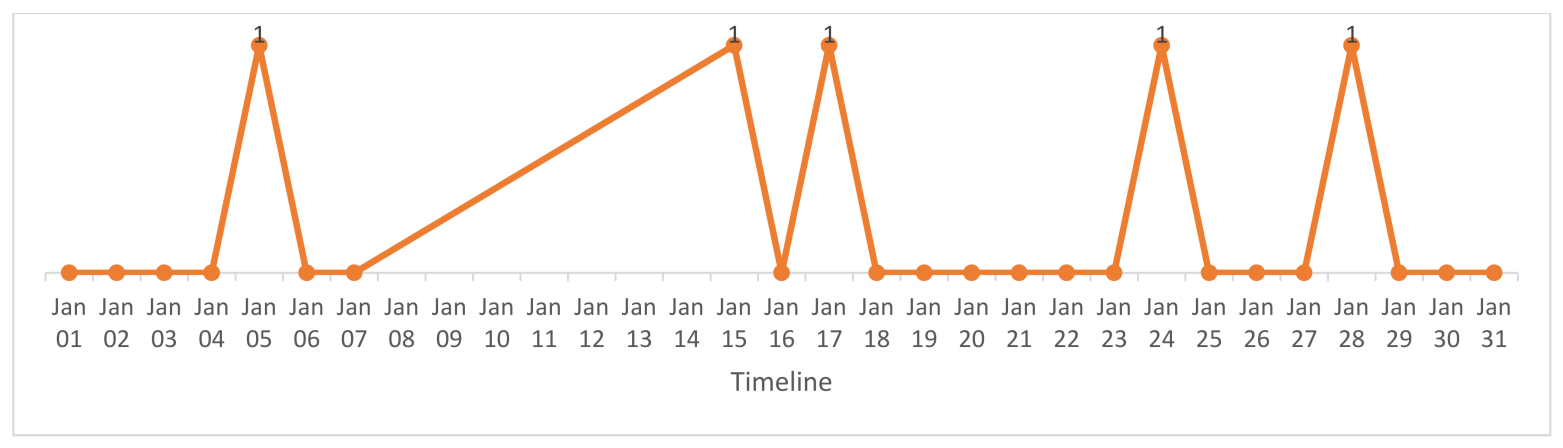
Epidemic curve

Three bloodstream infections were detected, two urinary colonizations were detected, of which one was also skin-colonized. The first case was detected on March 15, and the last on April 5. The age range was 18–29 years. By the end of the observation period, 1 patient was deceased and 4 were still hospitalized. Risk factors comprised a prolonged ICU stay, mechanical ventilation, central lines, broad-spectrum antimicrobial use including fluconazole, shared equipment including stethoscope, thermometer, glucometer, US probe, physiotherapy probe, and cuff manometer. Linelisting was done and the probable epidemiological link was pointing towards health care workers (HCW), shared equipment or environment. The epidemic curve constructed (Figure 1 (Fig. 1)) was suggestive of an intermittent source of exposure.

**Figure 2:**
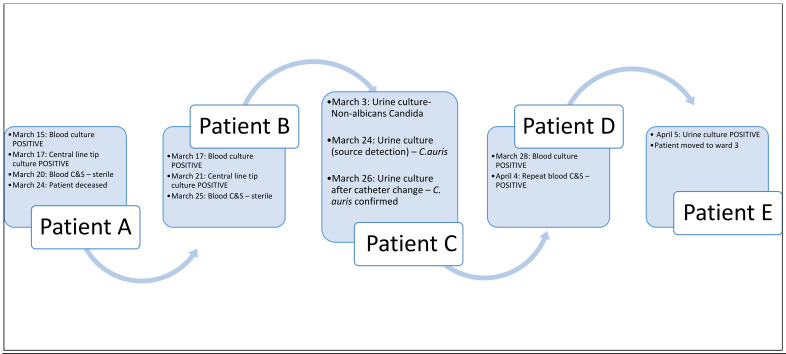
Timeline based on microbiological investigations and interventions

In Figure 2 (Fig. 2), the timeline based on microbiological investigations and interventions is summarized.

Axillary and groin swabs were taken from patients, and one patient was found to be colonized in the axilla. The same patient had urinary colonization with *C. auris* with no symptoms. In addition, the isolate obtained from another urine specimen was also not associated with clinical manifestations in the patient. Axillary and groin swabs from other patients yielded heavy growth of various Gram-negative isolates as pure and mixed growth. Screening of staff was not done due to the absence of consent. 

### Infection control measures

The following measures were implemented thoroughly:

- Immediate contact precautions for all identified patients,
- screening of all ICU contacts (axilla/groin swabs),
- hand hygiene adherence audits,
- daily proper cleaning with 1:50 household bleach (final dilution: 0.1% sodium hypochlorite) used 3 times daily in general cleaning and 1:10 household bleach (final dilution: 0.5% sodium hypochlorite) for terminal cleaning with a contact time of 10 minutes,
- chlorhexidine bathing of all patients,
- environmental surveillance,
- proper disinfection of shared equipment,
- temporary halt in admissions to ICU,
- re-education of staff on donning/doffing PPE and hand hygiene,
- standard and transmission based precautions advised in the ward along with cohorting in the ward to which the last patient was transferred.

Environmental surveillance yielded negative results for *C. auris*. Staff hand-hygiene non-adherence was noticed during outbreak visits; hence, staff hand-hygiene adherence was measured after taking awareness-reinforcing steps, resulting in improved compliance among doctors to 84%, nurses 87%, technicians/students 83% and grade-2 staff 76% after the intervention.

During source identification, a prior isolation of non-albicans Candida from the urine of patient C on 3 March 2025 was noted, necessitating species-level identification to assess the possibility of *C. auris*. Subsequent culture and MALDI-TOF identification confirmed this, thereby aiding in the identification of the index case. Furthermore, this patient’s skin was also found to be colonized. This suggests the possibility of patient C (admitted >1 month) being colonized with *C. auris* in urine and skin after prolonged fluconazole intake with spread of infection through non-adherence to hand hygiene protocols. Retrospective questioning showed that gloves were not changed after uro-bag emptying by grade-2 staff. 

Engagement with frontline healthcare workers and the medical officers in charge constituted a critical component of the outbreak investigation and resulted in the following key decisions necessitating and followed by immediate implementation for outbreak containment:

- Shifting existing patients to another ICU to enable deep cleaning and fumigation (indicated in the event of an outbreak) of ICU,
- temporary halt in admissions to ICU,
- proper cleaning and disinfection of all equipment,
- enhanced awareness of standard precautions with special focus on hand hygiene moments, hand hygiene steps, moments of glove change, proper daily cleaning of patients and chlorhexidine bathing,
- HCWs decolonization in case they were already colonized or subjecting them to a screening process,
- increase hand hygiene compliance and ensure adequate availability of hand rub, hand wash, and disinfectants in the critical care unit for the next 2 months,
- enable procurement of chlorhexidine solutions for decolonization of patients,
- reporting of Candida spp. even from the first urine sample, as opposed to the current protocol which requires isolation from a second specimen if patient is asymptomatic,
- decrease the number of medical/para-medical students in the ICU.

No new cases occurred 2 months after the implementation of infection prevention and control measures. 

## Discussion and conclusion

Non-albicans Candida isolates should be considered potential *C. auris* until accurately identified as another species. This highlights the critical importance of early organism identification and the implementation of alert systems. A prerequisite for outbreak control is the need for dedicated medical equipment in high-risk areas, regular refresher training for ICU staff with re-inforcement of hand hygiene awareness programs at specified intervals, the introduction of a checklist and audit tool to assist supervisory staff in monitoring and documenting environmental cleaning and disinfection surfaces and medical equipment, and finally the daily cleaning and decolonisation of the patient body. While sodium hypochlorite is effective at 1,000 ppm in >1 min and at 4,000 ppm in 1 min [1], there is currently no established protocol or evidence for antiseptic decolonization including use of 2% chlorhexidine digluconate [2].

## Notes

### Authors’ ORCIDs

- Hassan H: https://orcid.org/0009-0008-7090-372X
- Sathyadas A: https://orcid.org/0009-0002-1718-155X
- Reghukumar A:
https://orcid.org/0000-0002-5332-0978
- Sathyabhama MC:
https://orcid.org/0009-0001-7700-1373
- Jyothi R: https://orcid.org/0009-0000-0418-1265
- Manjusree S: https://orcid.org/0009-0000-5314-716X

### Funding

None. 

### Competing interests

The authors declare that they have no competing interests.
